# Conventional vs. Green Extraction Using Natural Deep Eutectic Solvents—Differences in the Composition of Soluble Unbound Phenolic Compounds and Antioxidant Activity

**DOI:** 10.3390/antiox11112295

**Published:** 2022-11-20

**Authors:** Milica Martinović, Nemanja Krgović, Ivana Nešić, Ana Žugić, Vanja Milija Tadić

**Affiliations:** 1 Department of Pharmacy, Faculty of Medicine, University of Nis, Boulevard Dr. Zorana Djindjića 81, 18000 Nis, Serbia; 2 Department for Pharmaceutical Research and Development, Institute for Medicinal Plant Research “Dr. Josif Pancic”, TadeusaKoscuska 1, 11000 Belgrade, Serbia

**Keywords:** natural deep eutectic solvents (NaDES), antioxidant tests, green extraction, ultrasound-assisted extraction, HPLC, soluble polyphenols

## Abstract

The aim of this study was to investigate the effect of the use of green solvents, natural deep eutectic solvents (NaDES), in comparison with conventional solvents on the extraction of free unbound phenolic compounds and the antioxidant activity of extracts of dried bilberry fruit, bilberry leaves and green tea leaves. After preparation of the extracts via ultrasound-assisted extraction using NaDES and conventional solvents (water and ethanol), spectrophotometric determination of total phenolic and flavonoid content, HPLC analysis of extracted polyphenols and antioxidant determination using FRAP, DPPH and ABTS assays were conducted. The results showed that NaDES have a great potential as agents for the extraction of phenolic compounds with potent antioxidant activity; the highest values of phenolic content and antioxidant activity were detected in the samples obtained by extraction using the NaDES combination betaine + urea. The bilberry leaves exhibited the highest flavonoid content among all extracts and turned out to be more active than bilberry fruits, to which they are often just a by-product during processing. The most active extract of all was the betaine-urea green tea leaves extract. Further research into the most active NaDES extracts should be performed.

## 1. Introduction

Phenolic compounds (PCs) are aromatic secondary plant metabolites divided into several different groups: phenolic acids, flavonoids, stilbenes, tannins and lignans, which participate in the physiology of the plants, their defence against pathogens and UV radiation and also contribute to morphological properties (i.e., PCs are responsible for the colour and bitterness of fruits) [1,2]. PCs are widely used in pharmaceutical, cosmetic and food industries, exhibiting many biological activities such as antioxidant, anti-inflammatory, antimicrobial, UV protective, anti-proliferative, cardioprotective, neuroprotective, anticancer activity, etc. [3,4,5,6,7]. Numerous studies have shown that PCs serve as natural, green alternatives to the medications used in prevention and treatment of some diseases [2,8].

Lately, it was reported that PCs existed both in free and bound form. While free—soluble PCs are easier to extract, the bound—insoluble PCs are linked to the cell wall structural components, such as proteins and carbohydrates, and they must undergo hydrolysis as a pretreatment before extraction. The bound PCs can be released in the gastrointestinal tract with the help of microorganisms and enzymes and then exert their effect, while free PCs can express their activity without any pre-treatment [9,10,11].

Recovery of PCs and other antioxidant compounds from plant materials is generally achieved through different extraction techniques. The conventional extraction methods, such as maceration, digestion, percolation, Soxhlet extraction, have been the most utilized techniques throughout the decades. However, they have some disadvantages, such as long duration of the process, low efficiency, requirement of large volumes of the extraction solvents, the unsuitability of some of the methods for thermolabile substances [12,13,14,15]. Therefore, some new, unconventional extraction techniques are being considered and becoming more and more popular, such as ultrasound-assisted extraction, microwave-assisted extraction, supercritical CO_2_ extraction, etc. [15]. These methods offer the possibility of higher extraction yields, shorter extraction time and environmentally friendly approaches, as well as reduced consumption of extraction solvents and less generation of toxic residues [12]. Ultrasound-assisted extraction (UAE) is a simple and low-cost method that is based on the generation of cavitation via ultrasound (frequency range 20–2000 kHz) [16]. Better extraction of PCs is enabled due to increased cell wall permeability and consequently increased diffusion of PCs into the extraction solvent [17]. According to our experience in our previous work, better extraction efficiency has been shown by ultrasound-assisted extraction, compared to traditional methods— maceration, percolation, Soxhlet extraction and digestion [18].

Traditionally used extraction solvents are water, methanol, ethanol, ethyl acetate, acetone, chloroform and *n*-hexane. They are used alone or in combination, depending on the polarity and chemical characteristics of targeted chemical substances [19]. Most of these organic solvents are volatile, flammable and are highly toxic for biocenosis and not degradable (not environment-friendly) [14]. Hence, natural deep eutectic solvents (NaDES), as new environmental-friendly solvents, are attracting significant attention. NaDES are composed of hydrogen bond donors (HBD) and hydrogen bond acceptors (HBA) of natural origin forming the stable systems via intramolecular hydrogen bonds [20,21]. Formed systems—eutectic solvents—firstly introduced by Abbot et al. [22] have lower melting point than each of the components, are non-flammable, non-volatile and biodegradable [23]. NaDES are shown to be especially efficient when it comes to extraction of PCs [24,25].

For this study, the following herbal drugs were chosen: bilberry fruits and leaves (*Myrtilli fructus*, *Myrtilli folium*, *Vaccinium myrtillus* L., Ericaceae) and green tea leaves (*Camelliae sinensis non fermentatum folium*, *Camellia sinensis* (L.) Kuntze, Theaceae). Selected plant materials are known for their high PCs content and good antioxidant activity [26,27,28]. While green tea leaves and bilberry fruits represent widely commercially used herbal drugs, bilberry leaves are mostly considered as waste products from mechanical harvesting of wild berries [29]. However, it was shown that they also can exhibit good antioxidant potential [27,30].

Taking into account the increasing awareness of the importance of environment-friendly raw materials and technological manufacturing methods, it has become necessary to undertake specific actions that might enable the reduction of the environmental impact of all processes involved in the research and production activities in the pharmaceutical and cosmetic industries [31]. Current trends show that the future is “green” [32]. The aim of the study was to perform the extraction of selected herbal preparations with the use of NaDES as “green” extraction solvents and UAE as a “green” extraction technique, followed by chemical characterization of the obtained extracts and determination of their antioxidant activity. The obtained results were compared to the ones acquired with conventional solvents (ethanol and water)”.

## 2. Materials and Methods

### 2.1. Plant Material and Reagents

Analytical grade reagents acetate buffer, 2,4,6-tripyridyl-s-triazine (TPTZ), HCl, FeCl_3_, Folin-Ciocalteu reagent, 2,6-di-*tert*-butyl-4-methylphenol (BHT), *n*-butanol (ButOH), acetone, ethyl acetate, sodium bicarbonate, 1,1-diphenyl-2-picrylhydrazyl(DPPH), 2,2′-azino-bis(3-ethyl-benzothiazoline-6-sulfonic acid) (ABTS), potassium persulfate (K_2_S_2_O_8_), malic acid, betaine, absolute ethanol (96%, *v*/*v*) and methanol were purchased from Sigma-Aldrich (St. Louis, MO, USA). Tartaric acid and urea were purchased from Centrohem (Serbia), sorbitol and glycerol were purchased from Comcen (Serbia) while citric acid was purchased from AnalarNormapur. Reference HPLC standards, gallic acid, protocatechuic acid, chlorogenic acid, hyperoside, epicatechin, epicatechin gallate, procyanidin B_2_, rutin, quercetin-3-*O*-glucoside, kaempferol-3-*O*-glucoside, delphinidin-3-*O*-glucoside, quercitrin, epigallocatechin, epigallocatechin gallate, cyanidin-3-*O*-galactoside and cyanidin-3-*O*-glucoside (purity ≥ 99%) were purchased from Extrasynthese (Genay, France).

Plant material used in this study represented the dried herbal parts of green tea leaves (*Camelliae sinensis non fermentatum folium*, *Camellia sinensis* (L.) Kuntze, Theaceae) and bilberry leaves and bilberry fruit (*Myrtilli folium*, *Myrtilli fructus*, *Vaccinium myrtillus* L., Ericaceae). The vouchers specimens (CSNonF_1121, VMF_1021 and VML_0921) were deposited at Herbarium of Faculty of Pharmacy, University of Belgrade (Belgrade, Serbia), where the identity confirmation was performed.

### 2.2. Preparation of NaDES

The process of NaDES preparation is based on the heating of the two individual components at 80 °C and their continuous stirring on magnetic stirring apparatus (IKAMAG, IKA, Verke, Staufen, Gerrmany) for 30–60min, until the mixture is melted and the clear liquid is formed [33]. In each mixture, the 30% (*v*/*v*) of distilled water was added. The following NaDES were prepared: tartaric acid + sorbitol, citric acid + sorbitol, betaine + urea and malic acid + glycerol (Table 1). All obtained NaDES were transparent colourless liquids.

### 2.3. Extraction

The extraction was carried out with NaDES and conventional solvents (distilled water and 50% ethanol, (*v*/*v*)) in a sonication water bath (Gesellschaft fur Labortechnik, Burgwedel, Germany), providing dried powdered plant material: solvent ratio of 1:20. The conditions of the UAE were set to 30 min at 50 °C. After the extraction, all samples were centrifuged (6000 rpm, 10 min) using laboratory Centrifuge LC 320 (Tehtnica, Slovenia), and supernatant was collected for further analysis (Table 2).

### 2.4. Total Phenolic Content Determination 

Total phenolic content (TPC) was determined by the slightly modified Folin–Ciocalteu method [34]: volume of 0.1 mL of the investigated extract was mixed with 0.5 mL of Folin–Ciocalteu reagent, after which 1.5 mL of sodium bicarbonate (20%) solution and 7.9 mL of distilled water wereadded to mixture. After 120 min at the room temperature (22 °C), absorbance was measured at λmax 765 nm using Evolution 60 UV/VIS spectrophotometer (Thermo Fisher scientific, Waltham, MA, USA). Gallic acid (0.02–0.1 mg/L) was used for calibration of a standard curve (equation of the calibration curve: y = 1.0983x + 0.0148, the linear regression at r^2^ > 0.99) and the results were expressed as mg of gallic acid equivalents per g of dry plant material (mg GAE/g DW-dry weight). The content of TP was presented as the mean of three measurements.

### 2.5. Total Flavonoid Content Determination

The content of flavonoids (TFC) was calculated using the modified aluminum chloride colorimetric method described by Woisky and Salatino [35]. The diluted standard solutions (0.5 mL) were separately mixed with 1.5 mL of 95% ethanol, 0.1 mL of 10% aluminium chloride, 0.1 mL of 1 M potassium acetate and 2.8 mL of distilled water. After incubation at room temperature for 30 min, the absorbance of the reaction mixture was measured at 425 nm with Evolution 60 UV/VIS spectrophotometer (Thermo Fisher scientific, USA). Rutin (0.05–0.5 mg/mL) was used as a standard for making calibration curve (equation of the calibration curve: y = 3.397x + 0.039, the linear regression at r^2^ > 0.99) and the results were expressed as mg of rutin equivalents per g of dry plant material (mg RE/g DW (dry weight)). The content of TF was presented as the mean of three measurements.

### 2.6. HPLC Analysis

For the purpose of polyphenol qualitative and quantitative analysis, an Agilent 1200 HPLC system equipped with photodiode-array (PDA) detector and Lichrospher 100RP 18e column (250 × 4.6 mm; 5.0 μm particle size) was used. Mobile phase contained 0.1 M phosphoric acid solution (phase A) and pure acetonitrile (phase B).

*Chromatographic conditions for anthocyanins analysis*. Gradient program was as follows: 0–11% B (5 min), 11–15% B (25 min), 15–18% B (8 min), isocratic 18% B (8 min), 18–30% B (4 min), 30–100% B (3 min), 100% B (7 min). Total run time was 60 min, flow rate 0.8 mL/min, injection volume 4 μL and column temperature 25 °C. PDA detector has been operating at 520 nm.

*Chromatographic conditions for flavonoids and phenolcarboxylic acids analysis*. Gradient program was as follows: 11–25% B (35 min), 25–40% B (20 min), 40–65% B (5 min), 65–100% B (10 min). Total run time was 70 min, flow rate 1.0 mL/min, injection volume 10 μL and column temperature 25 °C. PDA detector was set at 260, 280 and 325 nm.

All investigated extracts were diluted with deionized water to achieve concentration 25 mg/mL, and, before injection, were filtered using PTFE membrane filter.

Identification of compounds was based on the comparison of their retention times and UV-VIS spectra with those of standards. Once spectra matching succeeded, results were confirmed by spiking with respective standards to achieve a complete identification by means of the so-called peak purity test. Those peaks not fulfilling these requirements were not quantified. Quantification was performed by external calibration with standards. The concentrations of standards were: 0.52 mg/mL for protocatechuic acid, 0.45 mg/mL for chlorogenic acid, 0.40 mg/mL for hyperoside, epicatechin and epicatechin gallate, 0.36 mg/mL for procyanidin B_2_, 0.48 mg/mL for rutin, 0.39 mg/mL for quercetin-3-*O*-glucoside, kaempferol-3-*O*-glucoside and delphinidin-3-*O*-glucoside, 0.52 mg/mL for quercitrin, 0.45 mg/mL for epigallocatechin, 0.32 mg/mL epigallocatechin gallate, 0.42 mg/mL for cyanidin-3-*O*-galactoside and cyanidin-3-*O*-glucoside. The results were expressed as mg/g of dried drug weight.

### 2.7. Antioxidant Activity Determination

#### 2.7.1. DPPH Radical Scavenging Activity

The method adapted from Brand-Williams et al. [36] was used for performing DPPH assay. The assay is based on mixing 300 µL of test solution (the extracts diluted in methanol in 5 different concentrations) and 2.7 mL of 0.04 mg/mL freshly prepared methanol DPPH solution and recording the absorbance at 517 nm after 30 min incubation at room temperature in the dark, against methanol as a blank. The control solution (the mixture of methanol instead of test solution and DPPH solution) was used for calculating the free radical scavenging activity via the Formula (1):

DPPH radical scavenging capacity (%) = [(A_C_ − A_S_)/A_C_] × 100 (1)

A_S_ was absorbance of test solution treated with DPPH radical solution;
A_C_ was absorbance of control solution.

#### 2.7.2. Ferric Ion Reducing Antioxidant Power (FRAP Assay)

To determine the antioxidant power of the extracts, a slightly modified FRAP assay method by Benzie and Strain was used [37]. FRAP reagent was made by mixing 25 mL of 300 mM acetate buffer pH 3.6, 2.5 mL of 10 mM TPTZ (2,4,6-tripyridyl-s-triazin) solution in 40 mM HCl and 2.5 mL of 20 mM FeCl_3_ × 6H_2_O. For the purpose of performing the FRAP assay, 100 µL of different solutions, previously used in DPPH assay, and 3.0 mL of freshly prepared FRAP reagent were mixed. After 30 min incubation at 37 °C, the absorbance was recorded at 593 nm. The FRAP value was calculated from the calibration curve of FeSO_4_ × 7H_2_O standard solutions, covering the concentration range 100–1000 mmol/L (y = 0.777x − 0.0164, the linear regression at r^2^ > 0.99) and expressed as mmol Fe^2+/^g extracts. The spectrophotometric readings were conducted on Evolution 60 UV/Vis Spectrophotometer (Thermo Fisher scientific, Waltham, MA, USA).

#### 2.7.3. ABTS Radical Scavenging Activity

The ability of examined extracts to neutralize ABTS free radicals was evaluated by assay that Idris et al. described before [38]. The basic ABTS solution was prepared from 7 mM ABTS and 2.45 mM K_2_S_2_O_8_ water solutions, mixed in ratio 1:1 (*v*/*v*) and kept in the dark for 12–18 h, at room temperature. In order to obtain working solution, the basic solution was diluted with methanol, until the absorption value 0.700 was reached at 734 nm. Prior to assay, a series of samples dilutions was prepared. After that, 900 µL of working solution was added to 100 µL of diluted extracts, left in the dark for 7 min and absorbance was measured at 734 nm, against methanol as a blank. The control contained methanol instead of the extract. The percentage inhibition of ABTS radicals was calculated using the Formula (2):

ABTS radical scavenging capacity (%) = [(A_C_ − A_S_)/A_C_] × 100 (2)

A_S_ was absorbance of solution of the extract treated with ABTS radical solution;
A_C_ was absorbance of control solution.

All spectrophotometric readings, in antioxidant activity assays (DPPH, FRAP and ABTS), were also conducted for positive control (ascorbic acid) in the same manner.

### 2.8. Statistical Analysis

The statistical analysis was performed using IBM SPSS Statistics 20. One-way analysis of variance ANOVA was conducted for assessing the data, while Tukey’s test was used for posthoc analysis. Differences at *p* < 0.05 were considered as statistically significant. Pearson’s correlation test was used for calculating the correlation between obtained results of total phenolic and flavonoid content and antioxidant activity. Microsoft Excel 10 was used for creating Charts and Correlation Matrix.

## 3. Results

### 3.1. Total Phenolic Content

**Figure 1:**
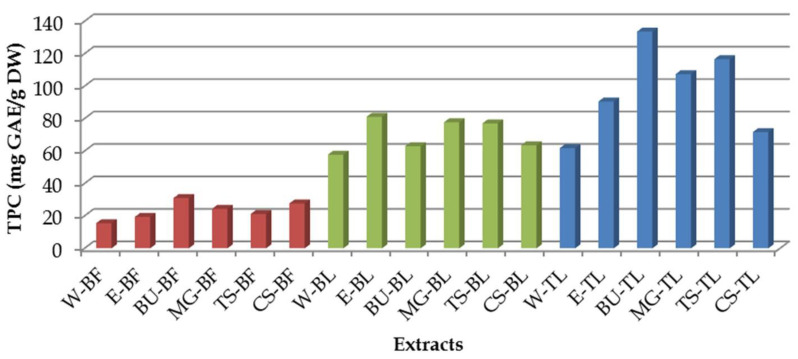
Total phenolic content (TPC) in the investigated extracts (the abbreviations are listed in Table 2).

The total phenolic content (TPC) in bilberry fruit, bilberry leaves and green tea leaves extracts is shown in Figure 1. The highest phenolic content was observed in green tea leaves extracts, while the lowest phenolic content was measured in bilberry fruit extracts. Although fluctuations among the obtained results were observed, the lowest amount of phenols was always measured in the water extracts (*p* < 0.05). In the case of bilberry fruit, the TPC value in NaDES extracts ranged from 21.15 to 30.94 mg GAE/g DW, with the highest value in the betaine-urea extract. The TPC values obtained from NaDES extraction were higher than the values obtained from water and ethanol extracts. When it comes to bilberry leaves, TPC values in NaDES extracts ranged from 63.51 to 77.64 mg GAE/g DW, with malic acid-glycerol and tartaric acid-sorbitol extracts showing the best extraction power. However, the ethanolic extract showed the highest TPC of all. In green tea leaves extracts, the TPC values for NaDES ranged from 71.66 to 133.55 mg GAE/g DW. Out of all the NaDES extracts, only the citric acid-sorbitol extract had lower TPC values than the ethanol extract, while the other NaDES extracts had significantly higher TPC values from both water and ethanol extracts of green tea leaves (*p* < 0.05). The highest phenolic content among green tea leaves extracts (also among all tested extracts, as well) was measured in the extract obtained by extraction using a mixture of betaine and urea with the addition of 30% water. The same trend was observed within bilberry fruit extracts, where also combination of betaine and urea was the most efficient in the extraction of phenols.

### 3.2. Total Flavonoid Content

**Figure 2:**
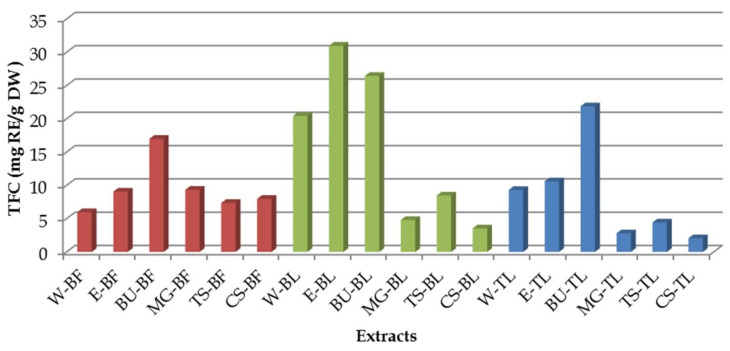
Total flavonoid content (TFC) in the investigated extracts (the abbreviationsare listed in Table 2).

The total flavonoid content (TFC) was also measured in the extracts obtained by extraction with conventional solvents (water and ethanol) and with NaDES (Figure 2). The highest numberof flavonoids was measured in bilberry leaves extracts, and among them the highest TFC was found in the ones prepared with ethanol (30.97 mg RE/g DW) and betaine-urea NaDES (26.45 mg RE/g DW). When water was used as solvent, it was shown that the TFC was significant in all investigated extracts (*p* < 0.05). Namely, the water extract had the lowest TFC in comparison to the rest of the used solvents only in the case of bilberry fruit extracts. Following the trend that the higher TPC was detected in extracts obtained using NaDES, among all prepared bilberry fruit extracts, the highest TFC was detected in betaine-urea extract. Similarly, the green tea leaves betaine-urea extract contained the highest numberof flavonoids. Contrary to the results obtained for TPC, in the rest of the investigated NaDES extracts, TFC wasdetermined to be smaller in comparison to the extracts obtained using the conventional solvents.

### 3.3. Chemical (HPLC) Analysis

**Figure 3:**
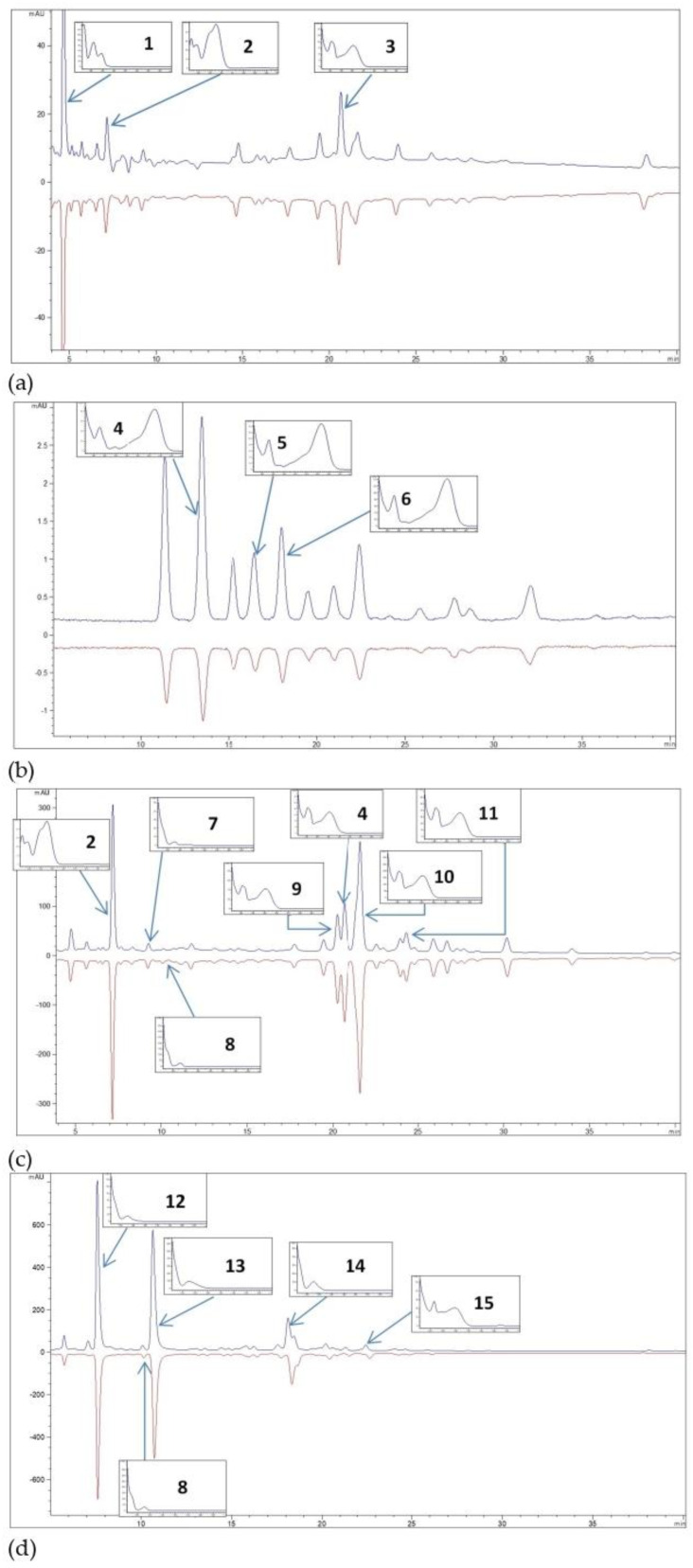
Representative examples of HPLC chromatograms of extracts obtained using NaDES−malic acid + glycerol (blue colour) and ethanol (red colour) of (**a**) bilberry fruit, polyphenol compounds profile (**1**, protocatechuic acid; **2**, chlorogenic acid; **3**, hyperoside); (**a**,**b**) bilberry fruit, anthocyanins profile (**4**, delphinidin−3−*O*−glucoside; **5**, cyanidin−3−O−galactoside; **6**, cyanidin-3-O-glucoside); (**c**) bilberry leaves; polyphenol compounds profile (**2**, chlorogenic acid; **4**, hyperoside; **7**, procyanidin B2; **8**, epicatechin; **9**, rutin; **10**, Quercetin−3−*O*−glucoside; **11**, quercitrin) and (**d**) tea leaves, polyphenol compounds profile (**12**, epigallocatechin; **8**, epicatechin; **13**, epigallocatechin gallate; **14**, epicatechin gallate; **15**, kaempferol-3-*O*-glucoside).

The results of the HPLC analysis of bilberry fruit, bilberry leaves and green tea leaves extracts prepared with conventional solvents and NaDES are presented in Figure 3 and Table 3.

Considering all plant extracts investigated in this study, it could be noticed that they contain a wide range of secondary metabolites. However, the main phenolic compounds include hydroxycinnamic acid (chlorogenic acid), hydroxybenzoic acid (protocatechuic acid), anthocyanins (cyanidin and delphinidin heterosides), flavonols (quercetin and kaempferol derivatives) and flavanols (epicatechin and epicatechin gallate esters).

In the case of bilberry fruit, the same phenolic profile was observed in all extracts, where the dominant compounds were protocatechuic acid (0.94–1.60 mg/g), chlorogenic acid (0.83–1.53 mg/g) and hyperoside (0.15–0.59 mg/g). Almost all NaDES extracts contained a higher amount of phenolics compared to extracts when conventional solvents were used. The combination of betaine-urea (1:2, mol/mol) and tartaric acid-sorbitol (1:2, mol/mol) were the most potent in PCs extraction. On the other hand, taking into account the extraction yields of anthocyanins (delphinidin-3-*O*-glucoside, cyanidin-3-*O*-galactoside and cyanidin-3-*O*-glucoside), NaDES that contained organic acids (tartaric, citric and malic) were more powerful in extracting these compounds than water, ethanol and BU.

Bilberry leaves extracts were the most abundant in chlorogenic acid (8.57–22.51 mg/g), followed by procyanidin B_2_ (7.57–18.59 mg/g) and quercetin-3-*O*-glucoside (4.88–9.58 mg/g), as well as other flavonoids (epicatechin, rutin, hyperoside and quercitrin). All NaDES was demonstrated to be more efficient solvents in comparison to water, and equal with ethanol, particularly malic acid-glycerol (1:2, mol/mol) and citric acid-sorbitol (1:2, mol/mol).

The chemical composition analysis of green tea leaves extracts revealed that the main active principles were epicatechin and kaempferol derivatives. Betain-urea (1:2, mol/mol), malic acid-glycerol (1:2, mol/mol) and citric acid-sorbitol (1:2, mol/mol) showed the highest extraction capacity for epigallocatechin (32.08–60.88 mg/g), epigallocatechin gallate (19.18–42.33 mg/g) and epicatechin gallate (5.63–17.79 mg/g) in comparison with conventional solvents. Epicatechin and kaempferol-3-*O*-glucoside were quantified in lesser amounts.

### 3.4. Antioxidant Activity

Three tests were used to measure the antioxidant potential—PPH, FRAP and ABTS assays. The results of the DPPH and ABTS assays are displayed via IC_50_ values, which means that the lower the value, the higher the antioxidant activity. With the FRAP test, the situation is reversed because the FRAP test is expressed through Fe^2+^ equivalents, so higher values indicate better antioxidant activity. All the results of testing the antioxidant activity of the prepared extracts from the bilberry fruit, bilberry leaves and green tea leaves are given in Table 4. All calculated antioxidant activities of the investigated extracts were muchlower than the positive control, ascorbic acid (Table 5).

According to all applied antioxidant tests, green tea leaves extracts showed the best antioxidant activity, while bilberry fruit extracts were the least active. The FRAP values ranged from 0.14 to 0.47 mmol Fe^2+/^g DW and 0.41 to 0.94 mmol Fe^2+/^g DW for the bilberry fruit and leaves extracts, respectively. When measured for the green tea leaves extracts, the FRAP values were in the range of 0.87 to 1.91. By far the highest FRAP value of all investigated extracts was shown for betaine-urea green tea leaves extract, which had the highest TPC content out of all the tested extracts. Both ABTS and DPPH tests confirmed that this extract is potentially the best antioxidant. On the other hand, NaDES citric acid-sorbitol extract of green tea leaves showed the lowest antioxidant activity, according to the results of all antioxidant tests. In the case of bilberry fruit extracts, the same trend was observed as in the case of TPC and TFC measurements, i.e., betaine-urea extract also had the strongest antioxidant activity. This was confirmed by both DPPH and ABTS assay. According to the performed assays, NaDES extracts betaine-urea and mallic acid-glycerol of bilberry fruit showed better antioxidant potential than conventional extracts. For bilberry leaves extracts, mixed results have been obtained. Namely, NaDESmallic acid-glycerol and tartaric acid-sorbitol extract of bilberry leaves exhibited the best antioxidant activity, according to the FRAP test. However, according to the DPPH test, the best antioxidant was the betaine-urea extract of bilberry leaves, while the ABTS assay revealed the greatest antioxidant potential of ethanol extract, followed by the betaine-urea extract. The obtained results had high correlation with the results obtained for flavonoid content in the extracts of this plant material.

### 3.5. Correlation Analysis

The results of the correlation analysis were portrayed in the correlation matrix within the Table 6, where red colour indicated the strongest correlation, both positive (r = 1) and negative (r = −1), while white colour designated no correlation (r = 0). The correlation analysis, which was performed to compare all measured parameters, revealed the strong correlation between the results of TPC, DPPH and FRAP, while the correlation between the DPPH and FRAP test results was inverse: lower values of DPPH corresponded to higher values of FRAP test. On the other hand, there was very little correlation between TPC and TFC scores. In addition, there was a statistically weak correlation between ABTS and FRAP tests results, while the correlation between ABTS and DPPH test results was very low. However, all three tests definitely revealed that the BU-TL extract had the best antioxidant activity.

## 4. Discussion

The principles of green chemistry are based on the cessation of the use of materials hazardous to humans and the environment and on the removal of hazardous substances from the synthesis and production process of chemical, pharmaceutical and cosmetic products [39]. Therefore, for extraction process, instead of using traditional extractants, some new solvents are being investigated, and currently one of the best options is NaDES. NaDES are also used for the production of new materials, separation of different types of analytes and in the fields of nanotechnology, biotechnology and bioengineering, etc. [40]. These eutectic systems are composed of two components associated via hydrogen bonds (HBA+HBD) of natural origin that are mostly plants’ primary metabolites (carbohydrates, organic acids, amines, amino acids, etc.) and normally have a biological role in a living organism [41]. Moreover, NaDES are eco-friendly, non-toxic, safe and biodegradable, have adjustable viscosity, low volatility, broad polarity range and good dissolving ability [42]. Besides all those advantages, NaDES applied within extracts can have beneficial effects per se due to their constituents. While ethanol is suspected to be carcinogenic for humans and ethanol extracts are not the best choice for the dermal application due to irritation and contact dermatitis [43], some NaDES can even have hydrating and nurturing effect on the skin. For instance, urea is a component of the natural moisturizing factor of the skin and can help preservation of skin integrity and even be useful in the treatment of some skin diseases [44]. The organic acids as α-hydroxyl acids can have numerous beneficial effects on the skin and are therefore used in the treatment of acnes or wrinkles [45,46].

So far, some studies have shown that NaDES can increase TPC and TFC compared to conventional solvents [47,48,49,50]. Yet, the high viscosity may be a limitation for using NaDES for extraction purposes, as mass transfer between the extraction solvent and the plant material may be limited [51]. Addition of water during the preparation of NaDES reduces the viscosity, increases polarity and helps the extraction of phenols. However, adding an enormous amount of water in NaDES can have a negative effect and cause weakening of the eutectic solvent structure [40]. Hence, in our study, 30% of water was added to the NaDES, which is in line with other studies [40,52,53,54,55].

In this study the obtained results revealed that all NaDES extracts had higher TPC yield compared to conventional water extract, while bilberry fruit extracts and green tea leaves extracts had higher TPC values compared to the extracts when conventional solvent ethanol was used. The results were mostly in agreement with those presented in the literature, in which dominantly choline-chloride-based NaDES were investigated since choline-chloride is probably the most used quaternium ammonium salt as an HBA for forming NaDES. Choline-chloride was also the first HBA used for preparation of eutectic solvent [22]. Choline-chloride-based NaDES were employed for microwave-assisted extraction of catechins from green tea leaves, and the best results were obtained when a combination of choline-chloride and lactic acid was used [56]. NaDES made of choline-chloride, glycerol and citric acid was shown to be an excellent extraction medium for anthocyanins from bilberry fruits [57]. The anthocyanins from bilberry peels, as main products from the fruit processing, were also extracted using choline-chloride-based NaDES via ultrasound- and microwave-assisted extraction processes. The obtained results were superior compared to the results obtained using conventional extraction techniques and solvents [58]. However, in our study, we applied some modification of HBAs. Taking into account that betaine-based eutectic systems were shown to possess better extraction efficacy than choline-chloride-based NaDES [59,60,61], we used this HBA in our investigation. Moreover, organic acids-based NaDES (especially based on malic acid) exhibited better antioxidant effect compared to choline-chloride=based NaDES [62]. To the best of our knowledge, so far, no studies were conducted on the green tea leaves, bilberry fruit and bilberry leaves using non-choline-chloride-based NaDES.

It is well known that different factors such as extraction solvent, temperature and sample-solvent ratio affect the extraction of phenolics from plant material [63]. Among them, the crucial one is the selection of a suitable solvent, as it was presented in our study. To estimate the effect of NaDES and conventional solvents on phenolic composition, HPLC analysis was applied. The bilberry fruit phenolics compounds analysis revealed the chemical profile being in line with the literature data [64]. Generally, BU solvent mainly enhanced protocatechuic acid and hyperoside extraction, while NaDES with organic acids enhanced the extraction of anthocyanins. The latter may be related to acidified polar solvents that favour anthocyanins extraction and improve their stability, as some authors reported [65]. In relation to the most abundant compound in bilberry leaves extracts, chlorogenic acid, CS and MG solvents showed better extraction efficiency than water and similar efficiency toethanol. Following the similar extraction trend, apart from chlorogenic acid, in these extracts several flavonoids (flavanols and flavonols derivatives) were also detected. Moreover, there is evidence that choline chloride-1,3-butanediol (1:2, mol/mol) solvent improved solubility of chlorogenic acid during extraction from bilberry leaves [66]. Compared to bilberry leaves phenolics extraction capability, CS and MG solvents also showed excellent extraction performance for the main active principles of green tea leaves, epicatechin derivatives (epigallocatechin, epigallocatechin gallate and epicatechin gallate) [67]. In the research of Jeong et al. [14], NaDES betaine-glycerol-glucose (4:20:1, mol/mol/mol) and 30% water extracted greater amount of epigallocatechin-3-gallate than 70% ethanol.

For in vitro testing of the antioxidant activity of various extracts, the best approach is considered to be the application of a combination of several methods based on different principles for determination of the antioxidant potential of the analysed material through different mechanisms of action. For assessment of the radical scavenging activity of the extracts investigated in this study, DPPH and ABTS assays were used since DPPH^•^ and ABTS^•+^ are relatively stable radicals that can interact with free radicals formed during lipid oxidation. DPPH assay is a rapid method based on measuring the absorbance at 517 nm of the solution where DPPH reagent is reduced by the antioxidant. The ABTS assay is based on the generation of a blue ABTS^•+^ that can be reduced by antioxidants. The FRAP test is carried out via the SET (single electron transfer) mechanism, involving no free radicals, and is based on the ability of antioxidants, soluble in water, to reduce iron from Fe^3+^ to Fe^2+^ [68,69]. In our study, the best correlation between TPC and antioxidant activity was observed with FRAP and DPPH assays, implying that these tests might be more reproducible and better reflect antioxidant properties than ABTS assay.

Interestingly, according to all used antioxidant assays, the bilberry leaves, as by-products of bilberry fruit processing, which are usually considered as a waste, have shown higher phenolic and flavonoid yields and better antioxidant activity compared to the fruits. The best antioxidant activity of all tested extracts was observed in betaine-urea green tea leaves extract.

High antiradical activity of the extracts may be a consequence of high phenolic content, responsible for scavenging the radicals. The PCs are particularly known for their antioxidant activity due to the presence of hydroxyl groups and their ability to donate an electron or hydrogen atom to the free radicals formed during oxidation, act as metal cation chelators and singlet oxygen quenchers [3,70]. In several studies, similar results were observed, namely that due to greater isolation of polyphenols when NaDES were employed, extracts showed a better antioxidant effect [50]. Our results pointed to the fact that the higher polyphenols content in plants extracts was a good indicator of their antioxidant activity.

## 5. Conclusions

This study offered insight into extraction potential of non-bound polyphenols using NaDES that were not based on choline-chloride as HBA. Among investigated plant materials (bilberry fruit, bilberry leaves, green tea leaves) the highest content of polyphenols and the highest antioxidant activity was observed in betaine-urea extract of green tea leaves. The combination of betaine and urea was shown to be a promising NaDES option since, among bilberry fruit extracts, betaine-urea NaDES showed the highest TPC and TFC yield and the obtained extract exhibited the best antioxidant activity. In addition, bilberry leaves were shown to have better antioxidant activity compared to the fruits. Moreover, bilberry leaves extracts expressed the highest flavonoid content among all investigated extracts. These results open possibilities for further investigations of the extraction of plant materials using NaDES, since the results of our research showed that extracts obtained by using green environmentally friendly NaDES solvents can have comparable or better antioxidant activity and higher content of phenolic compounds compared to those obtained by conventional extraction. All this confirms that NaDES solvents are a good alternative to traditional solvents for the preparation of extracts rich in bioactive components. Such extracts can be used further in the production of pharmaceutical and cosmetic products or in the food industries.

## Figures and Tables

**Table 1:** Natural deep eutectic solvents used for extraction.

Abbreviation	Component 1	Component 2	Mole Ratio
TS	tartaric acid	sorbitol	1:2
CS	citric acid	sorbitol	
BU	betaine	urea	
MG	malic acid	glycerol	

**Table 2:** Abbreviations of the investigated extracts.

Extraction Solvent	Bilberry Fruit	Bilberry Leaves	Green Tea Leaves
Water	W − BF	W −BL	W − TL
50% Ethanol	E − BF	E − BL	E − TL
Betaine + Urea	BU − BF	BU − BL	BU − TL
Malic acid + Glycerol	MG − BF	MG − BL	MG − TL
Tartaric acid + Sorbitol	TS − BF	TS − BL	TS − TL
Citric acid + Sorbitol	CS − BF	CS − BL	CS − TL

**Table 3:** Phenolic compounds identified by HPLC analysis in the investigated extracts. Means followed by a common letter are not significantly different at the 95% level of significance (*p* < 0.05).

	Bilberry Fruit					
	Water	Ethanol	Betaine + Urea	Malic Acid + Glycerol	Tartaric Acid + Sorbitol	Citric Acid + Sorbitol
Protocatechuic acid (mg/g)	0.94 ± 0.04 ^b^	1.03 ± 0.02 ^ab^	1.60 ± 0.15 ^a^	1.35 ± 0.15 ^ab^	1.48 ± 0.39 ^ab^	1.30 ± 0.28 ^ab^
Chlorogenic acid (mg/g)	0.86 ± 0.06 ^b^	0.83 ± 0.03 ^b^	1.40 ± 0.16 ^a^	1.47 ± 0.37 ^a^	1.53 ± 0.11 ^a^	1.51 ± 0.01 ^a^
Hyperoside (mg/g)	0.15 ± 0.03 ^b^	0.53 ± 0.03 ^a^	0.59 ± 0.03 ^a^	0.57 ± 0.06 ^a^	0.49 ± 0.01 ^a^	0.50 ± 0.07 ^a^
Delphinidin-3-*O*-glucoside (mg/g)	tr	tr	Tr	1.01 ± 0.05 ^a^	1.07 ± 0.02 ^a^	0.99 ± 0.05 ^a^
Cyanidin-3-*O*-galactoside (mg/g)	tr	tr	Tr	0.10 ± 0.03 ^a^	0.10 ± 0.04 ^a^	0.11 ± 0.03 ^a^
Cyanidin-3-O-glucoside (mg/g)	tr	tr	Tr	0.19 ± 0.02 ^a^	0.19 ± 0.01 ^a^	0.18 ± 0.01 ^a^
	**Bilberry Leaves**					
	**Water**	**Ethanol**	**Betaine + Urea**	**Malic Acid + Glycerol**	**Tartaric Acid + Sorbitol**	**Citric Acid + Sorbitol**
Chlorogenic acid (mg/g)	8.37 ± 0.06 ^c^	22.51 ± 0.50 ^a^	17.13 ± 0.21 ^b^	21.17 ± 1.75 ^ab^	19.48 ± 0.92 ^ab^	22.48 ± 1.57 ^ab^
Procyanidin B2 (mg/g)	nd	14.41 ± 0.80 ^a^	11.63 ± 0.85	18.59 ± 1.23	7.57 ± 1.16	15.77 ± 0.68 ^a^
Epicatechin (mg/g)	nd	1.92 ± 0.13 ^a^	2.19 ± 0.42 ^a^	1.61 ± 0.27 ^a^	0.51 ± 0.09	1.74 ± 0.08 ^a^
Rutin (mg/g)	1.93 ± 0.15	3.79 ± 0.12 ^a^	2.54 ± 0.13	3.24 ± 0.10 ^bc^	3.16 ± 0.05 ^c^	3.60 ± 0.26 ^ab^
Hyperoside (mg/g)	1.32 ± 0.11	3.31 ± 0.10	1.79 ± 0.09	2.57 ± 0.11	2.21 ± 0.07	2.85 ± 0.09
Quercetin-3-*O*-glucoside (mg/g)	4.88 ± 0.20	9.58 ± 0.22	6.03 ± 0.05	8.02 ± 0.08 ^a^	6.76 ± 0.16	8.29 ± 0.12 ^a^
Quercitrin (mg/g)	0.56 ± 0.07 ^de^	0.99 ± 0.10 ^a^	0.50 ± 0.02 ^e^	0.76 ± 0.05 ^bc^	0.69 ± 0.04 ^cd^	0.90 ± 0.06 ^ab^
	**Green tea leaves**					
	**Water**	**Ethanol**	**Betaine + Urea**	**Malic Acid + Glycerol**	**Tartaric Acid + Sorbitol**	**Citric Acid + Sorbitol**
Epigallocatechin (mg/g)	34.65 ± 1.55 ^b^	36.85 ± 0.59 ^b^	54.00 ± 1.11 ^a^	54.56 ± 0.49 ^a^	32.08 ± 0.84	60.88 ± 0.11
Epicatechin (mg/g)	6.66 ± 0.07	4.51 ± 0.07	9.02 ± 0.06	5.98 ± 0.11 ^a^	3.57 ± 0.06	5.84 ± 0.04 ^a^
Epigallocatechin gallate (mg/g)	nd	36.76 ± 0.67 ^a^	29.20 ± 1.31 ^a^	42.33 ± 0.58	19.18 ± 0.33	34.86 ± 1.58 ^a^
Epicatechin gallate (mg/g)	nd	12.18 ± 0.54 ^a^	17.79 ± 0.20	13.05 ± 0.43 ^a^	5.63 ± 0.16	9.97 ± 0.07 ^a^
Kaempferol-3-*O*-glucoside (mg/g)	nd	0.25 ± 0.05 ^ab^	0.76 ± 0.05	0.30 ± 0.03 ^a^	0.13 ± 0.00 ^b^	0.16 ± 0.01 ^b^

**Table 4:** Antioxidant activity of the investigated extracts.

	Bilberry Fruit					
	Water	Ethanol	Betaine + Urea	Malic Acid + Glycerol	Tartaric Acid + Sorbitol	Citric Acid + Sorbitol
FRAP (mmol Fe^2+/^g DW)	0.25	0.3	0.47	0.38	0.14	0.3
DPPH − IC_50_ (mg/mL)	4.47	3.24	1.05	1.64	2.38	2.43
ABTS − IC_50_ (μg/mL)	128.17	78.55	43.27	92.72	80.68	90.47
	**Bilberry Leaves**					
	**Water**	**Ethanol**	**Betaine + Urea**	**Malic Acid + Glycerol**	**Tartaric Acid + Sorbitol**	**Citric Acid + Sorbitol**
FRAP (mmol Fe^2+/^g DW)	0.41	0.66	0.82	0.94	0.94	0.51
DPPH −IC_50_ (mg/mL)	0.78	0.48	0.41	0.59	0.64	1.2
ABTS − IC_50_ (μg/mL)	49.49	15.56	23.75	29.82	39.38	26.57
	**Green Tea Leaves**					
	**Water**	**Ethanol**	**Betaine + Urea**	**Malic Acid + Glycerol**	**Tartaric Acid + Sorbitol**	**Citric Acid + Sorbitol**
FRAP (mmol Fe^2+/^g DW)	0.87	1.49	1.91	1.45	1.66	0.96
DPPH − IC_50_ (mg/mL)	0.42	0.24	0.09	0.28	0.25	0.44
ABTS − IC_50_ (μg/mL)	16.01	8.17	7.03	12.51	9.78	20.14

**Table 5:** Antioxidant activity of positive control (ascorbic acid).

	Ascorbic Acid
FRAP (mmol Fe^2+/^g DW)	15.94
DPPH − IC_50_ (μg/mL)	4.45
ABTS − IC_50_ (μg/mL)	2.31

**Table 6:** Correlation coefficient matrix.

TPC	1				
TFC	0.0161	1			
FRAP	0.9773	0.0973	1		
DPPH	−0.8460	−0.1724	−0.8519	1	
ABTS	−0.1965	−0.2045	−0.3545	0.1003	1
	TPC	TFC	FRAP	DPPH	ABTS

## Data Availability

Data are contained within the article.
